# Ten simple rules for developing good reading habits during graduate school and beyond

**DOI:** 10.1371/journal.pcbi.1006467

**Published:** 2018-10-11

**Authors:** Marcos Méndez

**Affiliations:** Area of Biodiversity and Conservation, Universidad Rey Juan Carlos, Móstoles, Madrid, Spain; Whitehead Institute, UNITED STATES

## Introduction

Scientific activity has been compared to a scholarly conversation [1]. As scientific communities are now very large, this conversation mostly proceeds by writing and reading. While lot of advice exists about the writing part [2–4], much less attention has been devoted to what to read (see, however, [5]). Reading is fundamental for keeping updated in the advances of your discipline. However, scholarly literature grows at an increasingly fast pace [6]. Scientists, regardless of the stage in their career, have trouble in navigating an overwhelming amount of relevant literature. This overload can potentially lead to increasingly selective or cursory reading. Consequences are detrimental for the training of new scientists by decreasing quality standards. Good reading habits are essential and need to be instilled from the very beginning of a scientific career. As a coordinator of a doctoral program, I have addressed this issue in a recent workshop, after realizing the demand of advice on this topic by many graduate students. Here, I summarize the content of this workshop in 10 simple rules for achieving good reading habits (see also [5]). They were mainly intended to guide early graduate students, but I believe they can also be useful at later career stages.

## Rule 1: Develop the habit of reading on a daily basis

A first step toward good reading habits is to realize that reading is a fundamental part of your training (or activity) as a researcher. If you do not read regularly, you will soon get out of date and will not be able to join the scholarly conversation. Piling or archiving unread papers (see Rule 9) only leads to a delusion of knowledge. Cursory or urgent reading when preparing a new article is neither efficient nor desirable. Instead, dedicate daily time slot for reading at least one paper. When to read is a very personal decision. I use my daily commuting time in public transport for that task; others start their working day doing some reading. You may find a particularly quiet moment in the day, either at work or at home. If you can only devote short time slots during the day and need several sessions to go through a paper, keeping a routine of daily reading is still advisable; find this time!

## Rule 2: Read thoroughly to build a sound background understanding of your topic

Particularly at early career stages, you need to get as much background information as possible. If you think you are short of time during your graduate period, just imagine how busy you will be later when you teach, supervise students, manage research projects; do not take shortcuts! Reading articles thoroughly will provide you a broad context about concepts, methods, results, and potential meanings and implications in your discipline. Once you have achieved a good background knowledge of a topic, you can start reading more selectively those papers or paper sections that fill a particular gap or curiosity. But a good background knowledge takes a long time to build; do your homework early in your career so that you can save time later.

## Rule 3: Do not ignore the pillars of your discipline; read the classics

I repeat, read the classics! You may be working at the cutting edge of science, but your discipline surely has a long history behind it. It is naïve to think that you can go on in science by reading only what is new, starting at the moment you got involved in science, and ignore past fundamental steps in the building of your discipline (even if obsolete for current standards!). You need to identify and read the foundational papers of your discipline, concerning concepts, methods, or views. If this brings you back two centuries, so be it! Only by reading the classics you will get a deep understanding of your research topic. This will not only prevent you from reinventing the wheel but will enable you to build a robust context for significant advances. Repeat, read the classics!

## Rule 4: If you have to get familiar with a new topic, consider reading in chronological order

Early in your career, or whenever you get into a new topic and gather a bunch of references to get a hint on the status of the field, consider reading them in chronological order. If all references are recent, this gives you an idea of how the conversation is developing and who is answering who. If the time lapse is longer, you will get surprised to discover how concepts, methods, or interpretations may have changed with time. Reading in chronological order will allow you to realize subtle—or dramatic—changes in term use or meaning that will profitably add to your background knowledge of the topic.

## Rule 5: Avoid narrow-mindedness by reading beyond your discipline

Interesting ideas, concepts, methods, or implications are waiting for you in the work of other disciplines. Keep an eye to advances in other fields. You do not need to become the next Leonardo da Vinci; find a balance between focused and broad reading. The trick is to identify a few journals with a broad scope or that provide reviews in a broad spectrum of topics within a larger field and browse their content regularly (see Rule 6). You may also join journal clubs or follow renowned scientists outside your specific field using social networks.

## Rule 6: Create a list of relevant journals

Find out which journals publish relevant information for your research and subscribe to the online alerts for new content. Think big; if you find yourself checking less than 20 journals, you are probably missing relevant sources. Include journals that only occasionally publish relevant information. Do not forget those journals in which new analytical, experimental, or statistical methods are published, as they not always overlap with the journals in which primary research is published. Be prepared to reassess your list of journals during your scientific career; journals bust and fade and editorial lines change.

## Rule 7: Not all interesting stuff will appear in articles; read books

Books distil accumulated knowledge or suggest groundbreaking ideas. Read books, both classic and recent. Spotting the classic books is relatively easy and, with luck, you may be allowed to “plunder” the library of the elders around (Rule 10). However, finding new relevant books is not as straightforward as finding new articles. Google Scholar and most of the big editorials offer alerts or mailing lists for new books that can be customized. Another possibility is to identify the journals that include regular sections on new books, such as *Quarterly Review of Biology*. Finally, check with the library of your institution how new books are advertised—probably as electronic alerts—as the traditional shelf with novelties is almost extinct.

## Rule 8: Use a reference manager to keep track of your literature

Reference managers [6, 7] will avoid chaotic accumulation of nonretrievable literature. Some discipline is required to avoid misuse of reference managers. First, devote some time every day to update your recent readings. Second, never store in your reference manager papers you have not read; scientific databases already do that for you. Unread papers are best filed in a “to read” folder, that can be subdivided according to topics or urgency. Third, remember that storing a paper in a reference manager forces you to identify meaningful key words. Thus, refrain from merely importing key words already included in the reference and create your personalized list of keywords because this will contribute to building your background knowledge of the discipline.

## Rule 9: Keep a long-term review for your own use as a way to remember what you read

The more you read, the more you forget! A fruitful way to remember what you read is to open one (or several) review(s) of a topic for your own use. You may use your reference manager to do that (Rule 8). Nevertheless, I recommend going a step further and have a spreadsheet or a text document to store basic information or main messages from the primary literature. Whatever the format you choose, this review will allow you to retrieve much more easily confirmatory or negative evidence that you might use when writing an introduction or a discussion. At the same time, it will serve as a "note to yourself" for future surveys in the topic. Of course, it can eventually lead to a formal review paper.

## Rule 10: Build your own library to make yourself independent and inspire others

As soon as you can, start your own collection of papers and books. Do not rely exclusively on your supervisor's (or your colleagues' or your institution's) library. Certainly, the internet is there to store the information for you …except when it is not! The same applies to the library of your institution, particularly outside the top scientific countries where research funding is haphazard [8, 9]. Consider subscribing to some particularly interesting journal or becoming a member of a society that publishes a relevant journal. Building your own library in paper might be challenging early in your career due to funding limitations or movement between institutions during your postdoc stage. Nevertheless, applying this rule should not wait until you get a permanent position, and a digital library is always an option. For scientists at later stages in their career, a personal library should be considered a must, as an important part of mentoring. I might be a little old-fashioned, but for those of us who grew intellectually surrounded by books and journals in our departmental library—and plundered others' libraries—the mere presence of books around creates an inspiring atmosphere. Provide the same atmosphere for your fellow students or colleagues.

## Final remarks

Good reading habits are essential at all stages of a scientific career. Senior scientists should cultivate them, caress literature, and instill these habits to their students as a part of their mentoring. In particular, Rule 10 can have an important part in the mentoring process as a way of giving example and as a tool for sharing knowledge and curiosity. A good deal of self-discipline underlies good reading habits, especially rules 1, 2, 3, 5, 8, and 9. As self-discipline is an important skill for early career scientists, they should not forget to include reading as a way to develop this skill. Finally, science is about the excitement of discovery and the amusement of enhanced understanding. Reading contributes to both; reading is, my colleagues, fun!
